# Stress and feelings in mothers and fathers in NICU: identifying risk factors for early interventions

**DOI:** 10.1017/S1463423619000021

**Published:** 2019-06-07

**Authors:** Chiara Ionio, Eleonora Mascheroni, Caterina Colombo, Francesca Castoldi, Gianluca Lista

**Affiliations:** 1 CRIdee, Dipartimento di Psicologia, Università Cattolica, Milan, Italy; 2 NICU, Ospedale dei Bambini V. Buzzi, ASST Fatebenefratelli-Sacco, Milano, Italy

**Keywords:** neonatal intensive care unit, parental stress, parenting, prematurity, risk factors

## Abstract

**Aims:**

The aims of this study were to explore parents’ stress levels and negative feelings after premature births and to identify the risk factors related to parents’ stress and negative feelings during their children’s neonatal intensive care unit (NICU) stay.

**Background:**

Preterm birth is a multi-problematic event that may put the babies in danger for both their medical and neurophysiological conditions and could have a negative impact on both the mother–father relationship and the parent–child interactions.

**Methods:**

The study involved 43 mothers and 38 fathers of preterm infants. All participants filled out the Parental Stressor Scale: Neonatal Intensive Care Unit and the Profile of Mood States.

**Findings:**

The results revealed significant differences between mothers’ and fathers’ responses to preterm births in terms of both stress and negative feelings. We found that, for mothers, their own young age and the baby’s need for respiratory support were significant predictors of stress; for fathers, their own young age and the baby’s lower gestational age and worse condition at birth were significant predictors of stress and negative feelings. The NICU may be a stressful place both for mothers and fathers. Identifying which mothers and fathers are at risk immediately after their children are born could help to direct specific interventions that can reduce these parents’ stress and prevent them from negative feelings.

The World Health Organization (1977) defined preterm birth as one that occurs before 37 weeks of gestation. Until the 1990s, prematurity was defined on the basis of birth weight; however, in recent years, gestational age (GA) has been considered the main indicator of preterm babies’ physical and neurological maturation (Sansavini and Faldella, 2013).

Preterm birth is a multi-problematic event that may present different consequences for baby’s development and for parents’ experience (Ionio *et al.*, 2016a; 2016b; 2017; Mascheroni and Ionio, 2018). It is well documented that preterm birth is associated with several poor outcomes in infants (especially for infants who have a GA of <32 weeks and those who weigh <1500 g) in various developmental domains, including motor, behavioural, cognitive, and sensory (Nepomnyaschy *et al*., 2012; Voigt *et al*., 2012; de Jong *et al*., 2015; Ionio *et al*., 2016b).

Preterm birth may have a negative impact on parents’ experience as well (Sameroff and Chandler, 1975). In fact, the premature birth of the baby suddenly interrupts the building of parents’ mental representations and expectations. Additionally, the possible critical condition both of the baby and the mother may shock parents, turning preterm birth in a highly stressful or traumatic events for mothers and fathers (Singer *et al*., 2003; Ravn *et al*., 2012; Monti *et al.*, 2013; Hoffenkamp *et al.*, 2015). Such births are often the unexpected results of mothers’ medical complications; these complications require immediate interruption of the pregnancies, including in emergency situations, to prevent serious threats to the health of the babies and mothers (Coppola and Cassibba, 2004). Such trauma can damage the new relationship between the parents and their babies and can be a risk factor in the mother–infant relationship, which in turn can have a negative impact on the infants’ future developmental outcomes (Wigert *et al.*, 2010; McManus and Poehlmann, 2012; Rogers *et al.*, 2013; Ionio and Di Blasio, 2014; Di Blasio *et al.*, 2017).

Different studies observed that premature birth generally interferes with transitions into parenthood and can be a potential risk factor for the construction of the parental function (Carter *et al.*, 2007; Nicol-Harper *et al*., 2007; Goodman *et al*., 2011; Murray *et al*., 2011). During the neonatal intensive care unit (NICU) stay, the adverse medical conditions of the baby prevent parents from immediately taking care of their newborn children (Feldman *et al*., 1999; Goldberg and DiVitto, 2005; Axelin *et al.*, 2010; Ionio *et al*., 2016a) and this may lead parents to experience higher level of stress, mostly because of parental role marginalization (Montirosso *et al*., 2012). In fact, when babies stay in the NICU, parents usually feel powerless, and they may be stressed and vulnerable to emotional difficulties (Clottey and Dillard, 2013; Sansavini and Faldella, 2013).

Preterm births can also interfere with parents’ everyday lives and routines. The parents spend many hours in the NICU, where they continue to experience the infant’s fragility (Clottey and Dillard, 2013) and live in a state of psychological and physical separation from their babies. This separation is aggravated by the artificial environment of the NICU, where in the days after the preterm delivery the medical staff – not the parents – used to take care of the infant’s well-being, often causing parents further pain and distress (Montirosso *et al.*, 2011; Sansavini and Faldella, 2013). In fact, initially, parents could only observe their infants and routine procedures. Only once the baby’s condition has improved, and if the parents felt ready, they start to be involved by nurses in the daily care of their infants (De Bernardo *et al*., 2017). The sense of powerlessness that parents may perceive in this situation can alter parental roles and further increase negative feelings such as anxiety, depression, anger, helplessness, and confusion (Müller-Nix and Ansermet, 2009).

Other studies focussed on the examination of potential risk factors that could influence parents’ reactions and feelings after the premature birth of their baby (Trombini *et al*., 2008). In particular, it was suggested that different demographic and clinical factors related to both parents and infants can influence parental stress levels and negative feelings in the NICU (DeMier *et al.*, 2000; Müller-Nix and Ansermet, 2009). The extent of the infants’ prematurity and the severity of their illness are predictors of parental stress. In fact, external infant characteristics are associated with immaturity and severity of medical status, which can in turn increase parental stress levels, thus interfering with the maternal and paternal roles (DeMier *et al*., 2000; Müller-Nix and Ansermet, 2009). It was also pointed out that babies’ neonatal characteristics, such as birth weight (<1500 g) and GA (<28 weeks), are related to parents’ levels of stress, depression, anxiety, and anger – both in mothers and in fathers (Dudek-Shriber, 2004; Franck *et al*., 2005; Zelkowitz *et al*., 2007; Chourasian *et al*., 2013; Alkozei *et al*., 2014; Woodward *et al*., 2014).

Moreover, demographic characteristics can also play an important role in predicting parents’ stress levels and negative feelings. Researchers have previously pointed out that stress levels and negative feelings in mothers are related to their education level, marital situation, partner-relationship quality, and perceived social support (Zelkowitz *et al*., 2007; Alkozei *et al*., 2014; Woodward *et al*., 2014; Di Blasio *et al*., 2015). Regarding paternal demographic characteristics, in previous studies, researchers have demonstrated that the presence of stress and negative feelings in fathers was related to both partner-relationship quality and perceived social support (Dudek-Shriber, 2004; Zelkowitz *et al*., 2007). However, other researchers have reported no association between stress levels and maternal demographics such as age, marital status, education, and employment status (Franck *et al.*, 2005; Chourasian *et al*., 2013).

Starting from these conflicting results, for this study, we wanted to better clarify which risk factors play important roles in predicting maternal and paternal reactions and feelings immediately after preterm births. As researchers have pointed out (Ionio *et al*., 2016a; Ionio *et al*., 2018), it is important to consider the characteristics and points of view of both mothers and fathers, as both parents are at risk after premature births. Moreover, in most of the past studies, researchers have focussed on experiences over the longer period of the infants’ hospitalizations; the analysed situations have already become normal for many of the parents. For this reason, it is also important to focus on parents’ experiences immediately after the premature births of their babies, such as their first experiences in the NICU, to increase knowledge about both mothers’ and fathers’ initial reactions.

In particular, one of our aims in the present study was to explore stress levels and negative feelings in both mothers and fathers immediately after the premature births of their babies. In addition, we wanted to identify which risk factors can further increase parental stress levels and negative feelings in the NICU and which can further damage the beginning of the parent–child relationship that occurs immediately after delivery.

Identifying these risk factors and the role that they play in parents’ reactions and feelings could help clinicians to better organize and administer interventions to reduce stress and negative feelings, before parents become symptomatic (Hynan *et al*., 2013).

## Methods

### Participants and procedure**s**

The current observational single-centre study was carried out from January 2013 to July 2015 in the NICU of the V. Buzzi Level III Hospital in Milan, Italy. This is the first part of a larger longitudinal study in which we tried to investigate the influences that mothers and fathers have on healthy preterm babies’ neuropsychological developmental outcomes from birth until preschool age.

The study was approved by hospital’s Research Ethics Committee, which required informed consent from each participant. Therefore, informed consent for the protection of privacy was a prerequisite to participate in the study. We recruited only Italian couple.

The inclusion criteria for newborns were GA <37 weeks, absence of congenital anomalies, and prenatally diagnosed brain lesions. Eligibility criteria for parents consisted of an age >18 years, no single-parent families, no manifest psychiatric or cognitive pathologies, and no drug addiction. The exclusion criteria for participation consisted of absence of informed consent.

All eligible parents were approached inside the NICU within 14 days of their infants’ births. During this period, parents were permitted to visit their baby for 10 h a day. The NICU’s rules consist of specified visiting hours. Both mothers and fathers can access the NICU from 11am to 9pm. Initially, parents can only observe their infants and routine procedures and only when they feel ready, they start to participate with nurses in the care of their infants.

During the first 2 weeks of infants’ NICU admission a research psychologist, introduced by the doctors of the ward, has presented the study to the parents, explaining that they would be involved in a longitudinal study that would investigate preterm children’s development from birth until preschool age.

If parents refused to participate (approximately 50% of the them, because they were totally focussed on their children), they were not enrolled in the study and no socio-demographic information was collected. If parents agreed to participate in the follow-up study, the researchers explained the content of the questionnaire to them. Parents who initially agreed usually did not change their minds after reading the questions, however, five fathers changed their mind after reading the questionnaires because they did not want to express their personal feelings. We asked the parents to complete the questionnaires separately to obtain answers from both mothers and fathers of the same couple.

A total of 43 mothers and 38 fathers of preterm infants were included. The sample’s characteristics are summarized in Table 1.

**Table 1:** Parental and infant characteristics

Parental characteristics	
Maternal age (years)	*M*=35.93; SD=5.99; range*=*21–49
Maternal level of education	
<High school	7.9%
High school	26.3%
>High school	65.8%
Paternal age (years)	*M*=39.91; SD=6.35; *range=*27–54
Paternal level of education	
<High school	38.7%
High school	12.9%
>High school	48.4%
Pregnancy characteristics	
Type of delivery	
Vaginal	5.7%
Caesarean	94.3%
Offspring order of the preterm	
First	41.2%
Second	52.9%
Third	5.9%
Multiple births	
Singleton	54.5%
Multiples	45.5%
Infant characteristics	
GA (weeks)	*M*=30.42; SD=2.79; range*=*24–35
Birth weight (grams)	*M*=1301.89; SD=432.62; range*=* 477–2200
1 min Apgar scores	*M*=6.27; SD=1.46; range*=*1–10
5 min Apgar scores	*M*=8.25; SD=0.86; range*=*6–10
Respiratory support (hours)	*M*=20.32; SD=25.52; range=0–95
Intubation	
Yes	27.5%
No	72.5%

*Note*: Sample size – mothers: *n*=43, fathers: *n*=38.

### Measures

#### Parental stress levels and feelings

We used the Parental Stressor Scale: Neonatal Intensive Care Unit (PSS: NICU; Miles, 2002; Italian version by Montirosso *et al.*, 2012) to measure stress levels in mothers and fathers. The PSS: NICU measures parents’ perceptions of the NICU. It is composed of 34 items and each item rated on a 5-point Likert scale (*not stressful* to *extremely stressful*). Items are divided into three subscales that measure stress caused by the machines in the NICU (*sights and sounds*), stress related to the infants’ status (*infant behaviour and appearance*) and stress related to the parents’ perceptions of their ability to become a caregiver (*parental role alteration*). This questionnaire showed good psychometric properties: the test–retest reliability ranged from 0.69 for the subscales to 0.87 for the total scale; the internal consistency ranged from 0.73 to 0.92 for the subscales and from 0.89 to 0.94 for the total scale. In the present study, this tool showed excellent psychometric properties, with Cronbach’s *α* reliability coefficients ranging from 0.93 to 0.98. The mean scores for each subscale and the mean overall scores were calculated.

### Profile of Mood States

We used the Profile of Mood States (POMS; McNair *et al*., 1971; Italian version by Farnè *et al*., 1991) to identify the presence of negative feelings in mothers and fathers. This questionnaire measures parents’ negative and positive mood states. It is composed of 58 items across six affective dimensions: *tension–anxiety*, *anger–hostility*, *fatigue–inertia*, *depression–dejection*, *vigour–activity*, and *confusion–bewilderment*. Previous studies demonstrated excellent psychometric properties: internal consistency (Cronbach’s *α*) in the subscales ranging from 0.63 (confusion–bewilderment) to 0.96 (depression–dejection) (Curran *et al*., 1995). In the current study, this tool showed excellent psychometric properties, with Cronbach’s *α* reliability coefficients ranging from 0.69 (confusion–bewilderment) to 0.93 (vigour–activity) for mothers and from 0.70 (vigour–activity) to 0.96 (depression–dejection) for fathers.

#### Parental and infant characteristics

We collected the following parental characteristics: age (years), type of delivery (vaginal or caesarean), number of child, type of delivery, offspring order, multiple births (multiple or singleton), and education level (less than high school, high school, or more than high school). We either recorded the information at the time of the assessment or obtained it through medical records.

We collected the following infant characteristics: GA (weeks), birth weight (grams), 1-min Apgar scores, 5-min Apgar scores, length of respiratory support (hours), and intubation (yes or no). We obtained this information from medical charts.

## Analysis

We analysed the data with SPSS Statistics 20.0. To compare maternal and paternal stress levels and negative feelings immediately after the premature births of their children, we used a paired sample *t*-test. Moreover, we performed a repeated-measure analysis of variance to compare the parents’ scores on both the PSS: NICU and POMS subscales.

We determined the extent to which particular factors influenced maternal stress and feelings by considering the following categories: (a) infant factors, such as GA, birth weight, 1-min Apgar scores, 5-min Apgar scores, length of respiratory support, and intubation; (b) pregnancy factors, such as type of delivery, offspring order, and multiple births; and (c) maternal demographic factors, such as age and level of education. We also determined the extent to which particular factors influenced paternal stress and feelings by considering the following categories: (a) infant factors, such as GA, birth weight, 1-min Apgar scores, 5-min Apgar scores, length of respiratory support, and intubation; (b) pregnancy factors, such as type of delivery, offspring order, and multiple births; and (c) paternal demographic factors, such as age, marital status, and level of education. We entered the factors into hierarchical linear regression models in the above-mentioned order, in separate blocks, and in a stepwise fashion; the PSS: NICU stress scores and POMS scores (total and for each subscale) were the outcome variables. We set the level of significance at *P*<0.05.

A post-hoc power analysis performed by G*Power demonstrated a power of 0.93 (*α*=0.05, large effect size of 0.35) for multiple regression analyses (Cohen, 1988).

## Results

### Maternal and paternal stress levels and negative feelings

We found significant differences between mothers’ and fathers’ stress levels, as measured by PSS: NICU. In particular, mothers obtained higher level of stress compare to fathers both in the three subscales (sights and sounds, infant behaviour and appearance, and parental role alteration) and in the total stress score. Considering the presence of negative feelings measured by POMS, we found that mothers had higher scores than fathers in the tension–anxiety and depression–dejection subscales. No further significant differences were found. These findings are summarized in Table 2.

**Table 2:** Descriptive statistics and paired *t*-test results for the study variables in mothers and fathers

	Mother’s score (*n*=43)	Father’s score (*n*=38)	
	*M* (SD)	*M* (SD)	*P*
PSS: NICU			
Total score	9.06 (2.46)	6.95 (1.91)	0.001
Sights and sounds	2.49 (0.73)	2.04 (0.523)	0.010
Infant behaviour and appearance	2.79 (1.01)	2.29 (0.740)	0.040
Alteration of parental role	3.78 (0.952)	2.64 (1.11)	0.000
POMS			
Total score	308.92 (44.42)	292.32 (41.85)	NS
Tension–anxiety	53.76 (11.92)	48.76 (9.81)	0.032
Anger–hostility	51.47 (12.02)	47.89 (12.32)	NS
Fatigue–inertia	54.45 (9.57)	51.03 (9.61)	NS
Depression–dejection	49.58 (8.74)	45.13 (8.40)	0.011
Vigour–activity	49.18 (11.02)	53.58 (8.65)	NS
Confusion–bewilderment	50.47 (11.35)	48.24 (10.81)	NS

Comparing the PSS: NICU maternal subscale, we found some significant differences (*F*=46.13; *P*<0.001). For mothers, stress levels related to alterations in the parental role were significantly higher than those related to either the infant’s behaviour and appearance (*P*<0.001) or NICU sights and sounds (*P*<0.001), and those related to the infant’s behaviour and appearance were significantly higher than those related to NICU sights and sounds (*P*=0.017). For fathers’ scores on the same scale, we also found significant differences; in particular, fathers’ stress levels related to alterations in the parental role were significantly higher than those related to NICU sights and sounds (*F*=4.89; *P*=0.016). We found no significant differences within the POMS subscales for either mothers or fathers.

### Predictors of stress levels and negative feelings

Significant predictors of both maternal and paternal stress levels are shown in Table 3. For mothers, the time that their babies spent on respiratory support was a significant predictor of stress related to NICU sights and sounds, and maternal age was a significant predictor of stress related to alterations of parental roles. In particular, younger mothers reported higher stress levels.

**Table 3:** Risk factors for maternal and paternal stress level measured by PSS: NICU

	*R* ^2^	*B*	*P*
Maternal stress level			
Sights and sounds			
Respiratory support	0.224	−0.013	0.044
Alteration parental role			
Respiratory support	0.391	−0.025	0.003
Maternal age	0.567	0.055	
Paternal stress level			
Sights and sounds			
Paternal age	0.395	−0.062	0.021
Infant behaviour and appearance			
1 min Apgar scores	0.344	−0.187	0.035
Alteration parental role			
5 min Apgar scores	0.372	−0.566	0.027
Total stress			
5 min Apgar scores	0.441	−1.00	0.021

*Note*: Sample size – mothers: *n*=43, fathers: *n*=38.

For fathers, paternal age was a significant predictor of stress related to NICU sights and sounds. In particular, younger fathers reported higher stress levels. Moreover, the 1-min Apgar score was a significant predictor of paternal stress related to infant and behaviour and appearance, and the 5-min Apgar score was a significant predictor for increase stress related to the alteration of parental role and total stress.

We found no significant predictors for the presence of negative feelings in mothers, despite what we found for fathers. As Table 4 shows, for fathers, 5-min Apgar scores, multiple births, and paternal age all significantly predicted tension and anxiety; GA was a significant predictor for both depression and anger; intubation, 5-min Apgar scores, and multiple births significantly predicted fatigue; and paternal age significantly predicted confusion.

**Table 4:** Risk factors for paternal presence of negative feelings measured by POMS

	*R* ^2^	*B*	*P*
Tension–anxiety			
5 min Apgar scores	0.251	−6.157	0.011
Multiple birth	0.374	−6.551	
Paternal age	0.549	−0.728	
Depression–dejection			
GA	0.350	−1.921	0.002
Anger–hostility			
GA	0.374	−2.866	0.001
Fatigue–inertia			
Intubation	0.515	11.469	0.000
5 min Apgar scores	0.646	−4.474	
Multiple birth	0.725	−6.837	
Confusion–bewilderment			
Paternal age	0.200	−5.126	0.025

*Note*: Sample size – mothers: *n*=43, fathers: *n*=38

## Discussion

Our first aim in this study was to explore stress levels and negative feelings in mothers and fathers immediately after the premature births of their children. From a clinical point of view, it is important to be aware of the feelings that parents experience when facing the difficulties and traumas surrounding the events that can arise during their children’s NICU stays after premature births. In fact, by paying attention to these difficult experiences, new parents could start a gradual process of transition to parenthood.

In previous studies on this topic, researchers have demonstrated that mothers, after their baby’s premature births and NICU stays, felt more stressed than their husbands did (Jackson *et al.*, 2003). Compared to fathers, mothers also experienced more negative feelings such as anxiety and depression (Zelkowitz *et al*., 2007; Lefkowitz, Baxt, and Evans, 2010). In agreement with previous research, our findings suggested that mothers had higher stress levels than fathers, probably because mothers usually spent more time in the NICU’s mechanical environment and felt more guilt about their infant’s impairment. It is also possible to infer that mothers are more afraid than fathers regarding their babies’ behaviour and appearance. Our findings also showed that fathers and especially mothers experience high stress levels related to alterations of the parental role. This seems to add further evidence to the fact that preterm births generally interfere with the transition into parenthood. As previously observed, the babies’ medical conditions prevent parents from immediately taking care of their newborn immediately after birth (Feldman *et al.*, 1999; Goldberg and DiVitto, 2005; Axelin *et al.*, 2010). As reported earlier, in the NICU where we conduct the study, initially, parents can only observe routine procedures on their infants; only once the baby’s condition has improved and when they feel ready, parents start to cooperate with nurses in the care of their infants. For this reason, we could infer that parents might feel powerless and helpless (Clottey and Dillard, 2013; Sansavini and Faldella, 2013) and, consequently, they might experience negative feelings such as anxiety, depression, anger, helplessness, and confusion (Müller-Nix and Ansermet, 2009). Moreover, as researchers have underlined, we found that mothers are more anxious and depressed than fathers (Zelkowitz *et al*., 2007; Lefkowitz *et al*., 2010).

Because these differences in terms of parents’ stress levels and negative feelings may depend on various risk and protective factors, in the present study, we also wanted to explore which factors cause stress and negative feelings in mothers and fathers after premature births. In the present study we would like to start to give further information to clinicians about parental risk factors. This attempt may be a very first step that could orient them to organize specific interventions for parents who have these particular characteristics so that they can deal with these events and support mothers and fathers as soon as possible.

Our exploratory findings suggest that preterm birth seemed to be particularly stressful for younger mothers and fathers. We can therefore hypothesize that for the younger parents in the current study, a lack of knowledge about what to do and what to expect provoked higher stress (Dudek-Shriber, 2004). Moreover, our results suggested that the mothers of infants who spent more time on respiratory support experienced more stress from the NICU environment. As researchers in previous studies have observed, the NICU can have a strong impact on mothers, such as by increasing their stress levels (Trombini, Surcinelli, Piccioni, Alessandroni, and Faldella, 2007). Fathers of children with lower GA and worse conditions at birth experienced higher stress levels and more negative feelings. These findings are in line with those of previous studies, in which extremely premature babies were shown to be physiologically unstable and less responsive to social interaction than were infants who were born later in pregnancy (Korja *et al*., 2012). In addition, extreme prematurity often resulted in a need for more medical equipment and a longer hospital stay to facilitate the infants’ development (Dudek-Shriber, 2004). Finally, it is important to underline that we did not find any factors that could predict the presence of negative feelings in mothers. As Lefkowitz *et al*. (2010) found, for mothers, the risk factors of loss of control, impairment of the normal routine, and fear of losing the child are more prominent than other factors; we did not investigate those factors in the present study.

In general, we could say that this study suggests the importance of paying attention to both parents’ experience in NICU immediately after the birth of their preterm child, and to factors that could potentially worsen this experience, leading parents to experience higher levels of stress and negative feelings. However, these results should also be read in the light of the limitations of this study. First, it is important to point out that our exploratory findings are taken from a limited sample size at a single NICU. An important next step would be to include more participants and new kinds of measures so as to better understand our results. Additionally, we used a sample of volunteer parents without detecting possible differences between parents that decided to take part in our study and parents that refuse to participate. Therefore, it may be possible that there is a selection bias. Participants’ responses may reflect their investment to better understand their situation. Further studies using a diverse and more representative samples will be necessary to better investigate the experience of those parents who do not decide to be assessed. Also, a longitudinal design would allow us to better understand whether factors such as parents’ stress and negative feelings after the preterm births of their children are linked with those children’s developmental outcomes.

In conclusion, our findings could be a starting point for helping primary care services to be more prepared to support parents following a preterm delivery in NICU. Raising awareness in practitioners involved in primary care services may help them to identify as soon as possible mothers and fathers who are at risk of severe responses and, consequently this could help them to provide adequate stress and negative feelings management interventions once they leave the hospital. As suggested by previous works, in order to prevent parents from experiencing stress and negative feelings after the premature births of their babies, it is essential to institute, as soon as possible specific interventions that reduce parental stress, decrease untoward responses, and improve both parental health and parenting behaviours (Maroney, 1994; Jackson *et al.*, 2003; Orapiriyakul *et al.*, 2007; Aagaard and Hall, 2008; Arnold *et al.*, 2013). Moreover, our findings may also help primary care services to be more prepared to give specific support to fathers of preterm babies (Lundqvist and Jakobsson, 2003; Pohlman, 2005; Candelori *et al*., 2017). In the current study we consider both the maternal and paternal points of view since we believe that both fathers and mothers of preterm infants equally need to be helped immediately after the childbirth and after the hospitalization period. Early family-centred interventions may be useful to prevent future difficulties in their relationship with infant and in child’s development (Ionio *et al*., 2018). Our results could stimulate nurses and other healthcare practitioners to take into account those factors that may impair the transition to parenthood after preterm birth, both for mother and for fathers. Particularly, our findings suggested that preterm birth is particularly stressful for younger parents that have preterm babies with low GA and that need respiratory support in the NICU. The current work may be a starting point to identify parents at risk. Further investigation will be necessary to better clarify which risk factors lead parents to experience higher level of stress and negative states that may be associated to the premature birth of their child.

## Financial support

The authors declare to have not received specific grant from any funding agency in the public, commercial, or not-for-public sectors.

## Conflicts of interest

The authors declare that they have no conflict of interest.

## Ethical standard

The study has been approved by the University Ethics Committee and have been performed in accordance with the ethical standards laid down in the 1964 Declaration of Helsinki and its later amendments.

## Informed consent

All parents gave their informed consent for themselves and for their children before their inclusion in the study.

## Authors’ contributions

All authors equally contributed in writing this manuscript. All the listed authors have reviewed and approved the final version of the manuscript as submitted.
